# Synergistic Effects of Omega-3 Fatty Acids and Physical Activity on Oxidative Stress Markers and Antioxidant Mechanisms in Aged Rats

**DOI:** 10.3390/nu17010096

**Published:** 2024-12-29

**Authors:** Zuzana Paduchová, Lívia Gajdošová, Barbora Katrenčíková, Martina Horváthová, Zuzana Országhová, Lucia Andrezálová, Jana Muchová

**Affiliations:** Institute of Medical Chemistry, Biochemistry and Clinical Biochemistry, Comenius University, Faculty of Medicine, Sasinkova 2, 811 08 Bratislava, Slovakia; zuzana.paduchova@fmed.uniba.sk (Z.P.); livia.gajdosova@fmed.uniba.sk (L.G.); barbora.katrencikova@fmed.uniba.sk (B.K.); martina.horvathova@fmed.uniba.sk (M.H.); zuzana.orszaghova@fmed.uniba.sk (Z.O.); lucia.andrezalova@fmed.uniba.sk (L.A.)

**Keywords:** aging, sarcopenia, omega-3 fatty acids, exercise, oxidative stress

## Abstract

Background: Aging induces degenerative processes in the body, contributing to the onset of various age-associated diseases that affect the population. Inadequate dietary habits and low physical activity are major contributors to increased morbidity during aging. This study aimed to investigate the combined effects of omega-3 fatty acid supplementation and physical activity on the markers of oxidative stress and antioxidant defense mechanisms in aged male Wistar rats (23–24 months). Methods: The rats were randomly divided into four experimental groups: a sedentary control (placebo, no exercise), a trained (placebo and moderate-intensity graded aerobic exercise; Ex), and two trained groups supplemented with low (160 mg/kg of body weight; O1 + Ex) and high (320 mg/kg of body weight; O2 + Ex) doses of omega-3 fatty acids. The biochemical and functional parameters related to sarcopenia and the markers of oxidative stress were measured in blood and gastrocnemius muscle. Results: The results demonstrated dose-dependent, synergistic effects of omega-3 fatty acid supplementation and physical activity. The higher dose (320 mg/kg of body weight) improved plasma antioxidant capacity (TEAC, +21.01%, *p* < 0.01) and GPx activity (+78.05%, *p* < 0.05) while reducing CAT activity in erythrocytes (−19.92%, *p* < 0.05), likely as an adaptive stress response. Combined interventions also normalized cholesterol levels, improved the functional parameters of sarcopenia (stride length, +14.82%, *p* < 0.001), and enhanced antioxidant protection in aged rats. Conclusions: These findings highlight the potential of combining omega-3 fatty acid supplementation and physical activity to counteract aging-related degenerative changes. Further research is needed to elucidate the underlying mechanisms and evaluate the long-term benefits of these strategies in aging populations.

## 1. Introduction

Aging is characterized by a progressive decline in physiological resilience, leading to impaired biological functions and homeostatic balance [1,2]. This process heightens vulnerability to stress, disease, and various injuries, ultimately contributing to age-related pathologies and increased mortality [3,4,5]. Recent studies have demonstrated that oxidative stress (OS) plays a central role in triggering cellular senescence through multiple pathways, including mitochondrial dysfunction, DNA damage, telomere shortening, lipid peroxidation, and chronic inflammation [6,7]. The relationship between OS and aging is complex, as OS not only accelerates cellular aging but also promotes chronic inflammation through senescence-associated secretory phenotypes, leading to immune system weakening [6].

Sarcopenia, a condition characterized by a progressive and generalized degenerative loss of skeletal muscle mass, quality, and strength during aging [8], significantly impacts the quality of life [9,10]. As increased levels of oxidative stress (OS) have been observed in aging muscles, reactive oxygen species (ROS) accumulation is thought to play a central role in sarcopenia pathogenesis [11,12]. Current research indicates that persistent OS not only accelerates the aging process but also induces various age-related diseases by damaging critical cellular structures [6]. Nutritional and lifestyle interventions have also emerged as strategies in the issue of sarcopenia and physical frailty [13,14,15] with potential links to cognitive impairment in old age [16,17].

Omega-3 fatty acids (FAs), particularly eicosapentaenoic acid (EPA) and docosahexaenoic acid (DHA), have been shown to enhance skeletal muscle mass by incorporating into membrane phospholipids, leading to increased muscle protein synthesis through the activation of the mTOR and p70s6k pathways, reduced protein breakdown via the inhibition of NF-kappa B signaling, and improved mitochondrial respiration [18,19]. For the positive effects of EPA and DHA to manifest, they need to be incorporated into the membranes of the cells involved in inflammatory processes, or into tissues such as skeletal muscle [20]. The bioavailability and efficacy of these FA are dose-dependent, requiring sufficient intake to induce meaningful changes in membrane composition [21]. Clinical research has established a threshold effect at 1.103 g/day of omega-3 FA intake, beyond which the impact on phenotypic aging tends to stabilize [22].

Similarly, physical activity has the potential to reduce the risk of oxidative stress-induced diseases and plays a crucial role in preventing sarcopenia [23]. Exercise induces ROS production, which in turn activates adaptive mechanisms [24,25], including the upregulation of endogenous antioxidant defenses through NRF2 activation and increased repair enzyme activity [26,27], thereby improving cellular homeostasis [28]. These effects not only enhance resistance to oxidative damage [29] but also trigger interleukin-6 production, initiating an inflammatory response while boosting anti-inflammatory cytokine levels [30].

This study represents a pioneering effort to compare systemic OS markers with those measured directly in muscle tissue following a combined intervention (omega-3 FA supplementation and physical activity). We aimed to investigate the effects of this combined approach using two distinct doses of omega-3 FA alongside physical activity on both the systemic (in blood) and tissue-specific (in muscles) markers of OS and antioxidant defense mechanisms. Our findings provide novel insights into the interplay between dietary supplementation and physical exercise in modulating OS responses at both systemic and local tissue levels.

## 2. Materials and Methods

### 2.1. Animals

Aged male Wistar rats (23–24 months, 500–600 g, *n* = 24) were obtained from the Centre of Experimental Medicine, Department of Toxicology and Laboratory Animal Breeding (Dobrá Voda, Slovak Republic). However, during the experiment, two rats died, one from the sedentary control and one from the trained group. Both deaths occurred during the third week of the intervention, and the necropsy revealed no pathological changes directly attributable to the intervention. These deaths were likely due to natural causes, given the advanced age of the animals. After a 10-day quarantine period, the rats were housed in groups of three in polycarbonate cages (50 × 36 × 19 cm) under controlled environmental conditions (23 ± 2 °C, 55 ± 5% humidity, 12 h light/dark cycle). Food and water were provided ad libitum. The study protocol was approved by the Ethics Committee of the Experimental Facility SK U 29016 in Bratislava and the State Food and Veterinary Administration of the Slovak Republic (Ro. 3054-/2021-220).

### 2.2. Experimental Design

The rats were randomly divided into four groups (*n* = 6 or 5 per group). The control group consisted of sedentary, aged rats receiving a placebo for 7 weeks. The trained group (Ex) received a placebo for 3 weeks, followed by 4 weeks of training exercise and continued placebo administration. Two intervention groups (O1 + Ex and O2 + Ex) received omega-3 FA supplements at 160 mg/kg and 320 mg/kg of body weight, respectively, daily for 3 weeks, followed by 4 weeks of omega-3 FA supplementation with treadmill exercise. The selected doses of 160 mg/kg and 320 mg/kg of body weight were based on the allometric scaling of commonly recommended human intakes of omega-3 FA (approximately 2 g/day and 4 g/day for a 70 kg individual) [31]. Omega-3 FA oil, administered orally after exercise using a micropipette, was prepared as a 1:1 mixture of DHA Finest Pure Fish Oil (Pharmax Ltd., London, UK) and Finest Pure Fish Oil (Vega Nutritionals^®^, Port Talbot, UK), supplied by Cultech Ltd. (Port Talbot, UK), achieving a DHA:EPA ratio of 1.5:1. Control and trained group (Ex) received sunflower oil as placebo. This oil primarily contains linoleic and oleic acid, with minor amounts of saturated fatty acids (palmitic and stearic acid), and lacks additional vitamins or bioactive compounds. Standard laboratory chow (KKZ-P/M, reg. no. 6147) was provided to all the groups. In line with Directive 2010/63/EU, we have adopted the 3Rs principle (Replacement, Reduction, Refinement). The 3Rs are now integral to the European and international scientific guidelines, including those from the ICH.

### 2.3. Exercise Protocol

The physical activity intervention comprised a graded aerobic exercise of moderate intensity, implemented through gradually increasing the load of exercise on a motorized rodent treadmill (47300 Rodent Treadmill NG, Ugo Basile, Italy). The exercise protocol involved 10 min daily sessions conducted 5 days per week over a 4-week period in a dimly lit environment to align with the nocturnal activity patterns of the rats. The 10 min duration was specifically chosen to accommodate the physiological limitations of the aged Wistar rats (23–24 months old), as prolonged exercise sessions can induce excessive stress and fatigue in this age group, especially in previously untrained rats [32,33]. To facilitate running, gentle electric shock stimuli were applied. The slightly modified protocol, adapted from Roya et al. [34], included a 1-week acclimation phase at a speed of 10 m/min on a flat plane. In the second week, the treadmill incline was set to 5°, and the speed was increased to 12 m/min. During the final two weeks, the incline was further elevated to 10° while maintaining a speed of 12 m/min.

### 2.4. Functional and Anthropometric Parameters

Stride length was measured the day before sacrifice using a paper-lined box (8.5 × 100 × 18.5 cm). To facilitate measurement, food coloring (brilliant blue) was applied to mark the rats’ paws, allowing for clear imprinting on the paper as the rats traversed the box. Stride length was defined as the distance between consecutive paw prints of the same foot.

Following sacrifice, body weight and the weights of three specific muscles (*soleus*, *gastrocnemius*, and *extensor digitorum longus*—EDL) were recorded. Muscle ratios were subsequently calculated as the percentage of each muscle’s weight relative to the total body weight.

### 2.5. Collection of Biological Material

Blood was collected after decapitation into EDTA-coated tubes and processed immediately. The samples were centrifuged at 1200× *g* for 10 min to separate plasma and blood cells. The plasma was aliquoted and stored at −80 °C, while the erythrocytes were hemolyzed, diluted 10-fold, aliquoted, and stored at −20 °C. The gastrocnemius muscle samples were flash-frozen in liquid nitrogen and stored at −80 °C until analysis.

### 2.6. Preparation of Tissue Homogenate

The gastrocnemius muscle (200 mg) was minced on ice and homogenized in a T-PER buffer (1:5, pH 7.6; Thermo Scientific, Dreieich, Germany) with a protease inhibitor cocktail (1:100, Sigma-Aldrich, Steinheim, Germany) using prefilled tubes containing 2.8 mm steel beads (Benchmark Scientific, Sayreville, NJ, USA). Homogenization was performed with a Bead Bug-6 homogenizer (Benchmark Scientific, Sayreville, NJ, USA) at a speed of 4350 rpm for 3 × 30 s cycles, interspersed with 30 s pauses. Following homogenization, the samples were centrifuged at 10,000× *g* for 5 min at 4 °C, and the resulting supernatant was aliquoted and stored at −80 °C. Protein concentration in the homogenates was quantified using the Pierce BCA assay kit (Thermo Scientific, Dreieich, Germany), with bovine serum albumin as a standard, and expressed in g/L.

### 2.7. Determination of Biochemical Parameters

Basic biochemical parameters were assessed at the accredited laboratory of Synlab Slovakia. The Alinity ci diagnostic device (Abbott, Abbott Park, IL, USA) was utilized, employing spectrophotometry to determine the concentrations of glucose, albumin, urea, total cholesterol, and triacylglycerol.

### 2.8. Determination of Total Antioxidant Capacity in Plasma and Muscle

The total antioxidant capacity of the plasma and muscles was measured using two established methods: trolox equivalent antioxidant capacity (TEAC) [35] and ferric reducing antioxidant power (FRAP) [36], with minor modifications. Trolox, a synthetic derivative of tocopherol, served as the standard for both assays. Antioxidant capacity was expressed in the plasma as µmol/L and in the tissue homogenates as either mmol/g or µmol/g of protein.

### 2.9. Determination of the Activity of Antioxidant Enzymes in Hemolysate of Erythrocytes

Antioxidant enzyme activities (SOD, GPx, and CAT) were measured in erythrocyte hemolysates. SOD and GPx were assessed using commercial kits (SOD determination kit, catalog No. 19160, Sigma-Aldrich, St. Louis, MI, USA, and Glutathione Peroxidase Assay Kit, catalog No. 703102, Cayman Chemicals, Ann Arbor, MI, USA, respectively), while CAT activity was assessed according to Bergmeyer [37]. Enzyme activities were normalized to hemoglobin content, which was quantified using the method described by Drabkin and Austin [38].

### 2.10. Western Blot Analysis of Antioxidant Enzymes Expression in Tissue Homogenates

The samples (20 μg of protein) were subjected to electrophoresis on 12% SDS-PAGE gels (55 V for 20 min, followed by 155 V for 90–120 min) [39]. After electrophoresis, proteins were transferred to polyvinylidene fluoride (PVDF) membranes using a semi-dry mini trans-blot cell (10 V, 60 min). The blotted membranes were subsequently blocked with 4% non-fat dry milk (Roth, Germany) in Tris Buffer Saline with Tween 20 and incubated for 1 h at room temperature. The membranes were then probed with primary antibodies specific for GPx-1/2 (1:800, Santa Cruz, CA, USA), CAT (1:500, Santa Cruz, CA, USA), and SOD (1:500, in 5% non-fat dry milk, Santa Cruz, CA, USA), along with glyceraldehyde 3-phosphate dehydrogenase antibody (GAPDH, 1:5000, Abcam, Cambridge, UK) as a loading control. A peroxidase-linked anti-mouse IgG (1:8000, Cell Signaling Technology, Danvers, MA, USA) or anti-rabbit IgG (1:8000; Abcam, Cambridge, UK) served as a secondary antibody. Protein bands were visualized using a chemiluminescence substrate (Clarity^TM^ Western ECL Substrates, BioRad, Dubai, United Arab Emirates) in a 1:1 ratio for 5 min. The protein expression levels were analyzed using the ChemiDoc XR system, and corresponding band intensities were quantified using the ImageLab software (version 5, BioRad, Dubai, United Arab Emirates).

### 2.11. Determination of Thiobarbituric Acid Reactive Substances (TBARS) in Muscle

The muscle TBARS levels were measured in the tissue homogenates using a modified method by Ohkawa et al. [40]. A 100 µL of 20% homogenate was mixed with 2% H_3_PO_4_ and 0.67% thiobarbituric acid, and then incubated at 95 °C for 30 min before cooling on ice for 10 min. After adding n-butyl alcohol, the mixture was centrifuged at 10,000 rpm for 15 min. The absorbance of the supernatant was measured at 532 nm, and TBARS levels were quantified using a 1,1,3,3-tetramethoxypropane standard curve (0–30 µmol/L) and expressed as µmol/g of protein.

### 2.12. Determination of Advanced Oxidation Protein Products (AOPP)

AOPP levels were determined in plasma using the spectrophotometry method according to Witko-Sarsat et al. [41]. A 30 μL of precipitating agent (1 mol/L MgCl_2_·6H_2_O and 2% dextran sulfate, mixed in a 1:1 ratio, was added to 150 μL of the plasma samples, followed by overnight incubation at 4 °C. Lipids were removed via centrifugation (3000 rpm, 20 min). The supernatant was diluted with PBS (pH 7.4), and 100 μL of the sample and 10 μL acetic acid were added to a microtiter plate. Chloramine T and PBS served as calibration and blank, followed by KI (5 μL of 1.16 M) and acetic acid (10 μL). Absorbance was measured at 340 nm, and the results were expressed in μmol/L.

### 2.13. Determination of 8-Isoprostanes (8-isoP)

8-isoP levels were determined in the plasma using a commercial EIA kit (catalog No. 516361, Cayman Chemicals, Ann Arbor, MI, USA). The amount of bound conjugate was measured at 405 nm and inversely correlated with the concentration of 8-isoP in the sample and expressed in pg/mL.

### 2.14. Determination of Lipid Peroxides

Lipid peroxide levels in the plasma were measured using a spectrophotometric method based on the conversion of iodide to iodine [42]. The concentrations of lipid peroxides were expressed in nmol/L.

### 2.15. Statistical Analysis

Data analysis was performed using the GraphPad InStat Software version 3.06 (GraphPad Software, Boston, MA, USA). Data are presented as median and quartiles (Q1 and Q3) or mean and standard deviation (SD). Normality was tested using the Shapiro–Wilk W-test. The Kruskal–Wallis test with Dunn’s post hoc analysis was employed for comparisons against the sedentary controls. For the protein expression of the antioxidant enzymes in the muscle, one-way ANOVA with Tukey–Kramer post hoc test was applied. A significance level of *p*-values < 0.05 was established for all the statistical tests.

## 3. Results

The effect of a combined intervention using omega-3 FA alongside physical activity on the markers of oxidative stress and antioxidant defense in the aged rats was studied. Table 1 and Table 2 summarize the functional, biochemical, oxidative stress, and antioxidant parameters in both the sedentary and trained aged rats, highlighting the impact of combining omega-3 FA with physical activity on these parameters. Table 1 focuses on the plasma and erythrocyte parameters, while Table 2 details the findings related to the gastrocnemius muscle.

In the group of rats subjected to forced exercise (Ex), a statistically significant increase was observed in stride length (+9.27%; *p* < 0.05) and the EDL muscle mass index (+15.30%; *p* < 0.05). Physical activity also significantly elevated erythrocyte GPx activity (+20.41%; *p* < 0.05) and plasma total antioxidant capacity, as measured by the TEAC method (+13.73%; *p* < 0.05). Conversely, a significant decrease in the total antioxidant power was noted using the FRAP method (−24.93%; *p* < 0.05). Additionally, physical activity led to a notable reduction in the TAG levels (−30.82%, *p* < 0.05) (Table 1). The impact of physical activity on gastrocnemius muscle parameters was evident only in terms of oxidative damage to lipids, as indicated by a significant decrease in TBARS (−29.58%, *p* < 0.05) (Table 2).

Two different doses of omega-3 FA were investigated: a lower dose of 160 mg/kg body weight and a higher dose of 320 mg/kg body weight. The combined intervention involving the lower dose of omega-3 FA (O1 + Ex) and physical activity significantly reduced erythrocyte CAT activity (−22.00%; *p* < 0.05) while simultaneously increasing stride length (+12.85%; *p* < 0.05) (Table 1). However, no significant effects on muscle parameters were observed with this intervention (Table 2).

**Figure 1:**
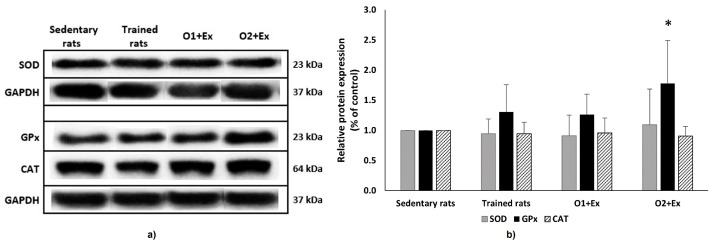
Relative expression of antioxidant enzymes in gastrocnemius muscle. (**a**) Representative Western blot analysis of the antioxidant enzyme protein expression (SOD, GPx, and CAT) in the gastrocnemius muscle of sedentary and trained rats (Ex, O1 + Ex, and O2 + Ex). Western blot images are representative of independent biological replicates (*n* = 5 for SOD; *n* = 3 for GPx and CAT). Multiple bands originated from the original image of the same sample. GAPDH served as a loading control. (**b**) Semi-quantitative evaluation of protein expression normalized to GAPDH. Data are presented as mean ± standard deviation (SD). *Statistically significant values are defined as *p* < 0.05 (ANOVA with Tukey–Kramer post hoc test). SOD—superoxide dismutase; GAPDH—glyceraldehyde 3-phosphate dehydrogenase.; GPx—glutathione peroxidase; CAT—catalase; trained rats (Ex)—supplementation with a placebo daily for 3 weeks, followed by 4 weeks of placebo intake along with daily exercise for 10 min; O1 + Ex—supplementation with omega-3 FAs in a dose of 160 mg/kg of body weight/daily for 3 weeks, followed by 4 weeks of omega-3 FA intake in the indicated dose with daily exercise for 10 min; O2 + Ex—supplementation with omega-3 FAs in a dose of 320 mg/kg of body weight/daily for 3 weeks, followed by 4 weeks of omega-3 FA intake in the indicated dose with daily exercise for 10 min.

In contrast, the combined intervention with the higher dose of omega-3 FA (O2 + Ex) significantly increased stride length (+14.82%; *p* < 0.001). Additionally, this group showed a decrease in erythrocyte CAT activity (−19.92%; *p* < 0.05) and an increase in plasma TEAC (+21.01%; *p* < 0.01) (Table 1). We also determined the relative expression of antioxidant enzymes in muscle gastrocnemius by the Western blot analysis. We found out that the treatment significantly upregulated GPx expression by 78.05% (F = 3.069, *p* = 0.0527; post hoc Tukey–Kramer test, *p* < 0.05) (Figure 1). SOD expression showed a trend toward an increase, further supporting a targeted effect on muscle antioxidant defense. Overall, these findings suggest that the combined intervention with omega-3 FA and exercise has a positive impact on both physical performance and antioxidant defense mechanisms in muscles.

## 4. Discussion

This study provides a novel comparative analysis of the systemic and muscle-specific oxidative stress markers in aged Wistar rats subjected to a combined intervention of omega-3 FA supplementation and physical activity. We investigated the effects of this combined intervention on selected parameters related to the aging process, focusing primarily on oxidative stress markers. The results demonstrate a positive synergistic effect of these interventions, particularly at higher omega-3 FA doses, on stride length and plasma antioxidant capacity, accompanied by a concurrent increase in muscle relative GPx expression. The combined intervention also normalized the total cholesterol levels and reduced erythrocyte CAT activity, while producing tissue-specific changes in the muscle antioxidant capacity and increased concentrations of lipid oxidative stress markers in plasma.

Aging significantly impacts structural integrity and energy metabolism, with muscle tissue loss being a key sign [43,44]. Changes in body composition, such as reduced muscle mass and stride length, are the hallmark indicators of sarcopenia [45] commonly observed in aged animals [46,47,48]. In our study, physical activity increased stride length and EDL muscle ratio in the aged rats, while combining exercise with omega-3 FAs—especially at higher doses—further enhanced stride length. These findings suggest that physical activity promotes muscle growth. While muscle growth was traditionally associated with resistance training, research has shown that aerobic exercise can also induce skeletal muscle hypertrophy. This occurs through alterations in protein metabolism, suggesting that various forms of physical activity contribute to muscle development [49]. This effect is amplified by omega-3 FAs, which support muscle anabolism and reduce inflammation [18,50,51,52]. Fish oil supplementation improved muscle strength and function in the aged rats, suggesting its potential to counteract age-related muscle decline [53]. Furthermore, omega-3 FA supplementation improved the insulin signaling pathways and enhanced the expression of the genes involved in glucose uptake and metabolism in skeletal muscle, indicating a positive effect on muscle health [54]. The beneficial effects are partly mediated through EPA and DHA metabolites, including resolvins and protectins, which provide anti-inflammatory and tissue-protective effects [55].

We analyzed the plasma biochemical parameters to assess age-related changes in metabolism and the impact of interventions. Our findings indicate that total cholesterol levels, which tend to increase with age due to impaired metabolism [47,56,57], exceeded the physiological range (1.57–2.59 mmol/L). This elevation may reflect reduced bile acid biosynthesis [58] or age-related growth hormone decline [59,60]. Omega-3 FAs, particularly EPA and DHA, have been extensively studied for their effects on lipid profiles, including TAG and total cholesterol [61,62,63]. Notably, the combined intervention effectively normalized cholesterol levels back into the physiological range.

Additionally, the plasma TAG levels were markedly elevated above the physiological range (0.36–0.88 mmol/L) across all the groups, aligning with previous reports documenting increased TAG levels in aged rats [64]. While both exercise [65] and omega-3 FA supplementation independently lower TAG levels [61,66,67], we expected that combined intervention would result in an additional TAG-lowering effect; however, our results did not confirm that. Our results showed that exercise alone significantly reduced TAG levels, while the combined interventions did not produce additional effects on TAG, corroborating the findings from studies in rats [68] and mice [69,70].

Urea levels remained within the physiological range (4.39–7.18 mmol/L), except in the rats receiving the higher omega-3 FA dose with exercise, where the levels slightly exceeded this range. Contrary to previous findings [71], neither exercise nor low-dose omega-3 FA interventions resulted in a significant reduction in urea concentrations.

Aging is frequently associated with increased OS [72,73,74]. We assessed several OS markers, including lipid peroxidation (LPx, 8-IsoP, and TBARS) and protein oxidation (AOPP), alongside plasma antioxidant capacity and the activity of antioxidant enzymes (SOD, GPx, and CAT) in the blood, and enzyme expression in the muscle tissues. Our results indicated that physical activity significantly increased GPx activity in the blood, while its expression in the muscles was only slightly elevated. This suggests that the primary effect may occur at the systemic level, rather than within muscle tissues [75]. It could be explained by enhanced erythrocyte resistance to oxidative damage after regular exercise [76].

While OS markers like AOPP, 8-IsoP, and LPx showed non-significant decreases, the TBARS levels in the muscles significantly declined, consistent with the findings by Pinho et al. [77]. Decreased levels of TBARS in muscle tissues after low-intensity exercise can be attributed to several mechanisms, e.g., enhanced antioxidant defenses, improved blood flow, adaptations to oxidative stress, modulation of inflammation, and changes in fatty acid composition. These mechanisms work synergistically to protect muscle cells from oxidative damage and maintain cellular integrity during and after physical activity [78,79,80]. However, the literature reports inconsistent results regarding OS markers, including increased MDA levels following acute exercise [81]. Antioxidant assessments in plasma yielded mixed results: the TEAC values significantly increased, likely due to thiol-group antioxidants, while the FRAP values decreased, potentially reflecting the depletion of low-molecular-weight vitamins such as C and E [82,83]. Notably, the albumin levels, the most abundant protein in the plasma, did not significantly change due to the interventions. These findings underscore the distinct mechanisms underlying the TEAC and FRAP assays. The increased TEAC levels observed may be attributed to enhanced GPx activity, potentially mediated by the activation of the nuclear factor (erythroid-derived 2)-like 2 (Nrf2) pathway involving the upregulation of antioxidant enzymes (e.g., SOD, GPx, and others) or glutathione (GSH) synthesis [84,85].

In the group receiving a combined intervention with a lower dose of omega-3 FA, we observed a significant reduction in CAT activity in the erythrocytes, despite some studies suggesting that omega-3 FAs can enhance CAT activity [86,87]. The potential mechanism of omega-3 supplementation includes a direct increase in CAT levels within peroxisomes and cytoplasm or the replacement of polyunsaturated fatty acids in cell membranes that have been damaged by free radicals [86]. However, there was a non-significant increase in the 8-isoP levels. No significant changes were detected in the gastrocnemius muscle. Interestingly, the higher dose of omega-3 FAs combined with physical activity produced different effects on the measured parameters. CAT activity was significantly reduced, while the total antioxidant capacity of the plasma, assessed using the TEAC method, increased. This could be explained by the different Michaelis constants (Km) of the enzymes (CAT and GPx) for hydrogen peroxide, their substrate. Each enzyme efficiently catalyzes the removal of H_2_O_2_ within its respective in vivo substrate concentration range [88]. Lipid oxidation markers in the plasma (8-IsoP and LPx) were slightly elevated; however, the TBARS levels decreased in the muscles, but not significantly. Additionally, GPx protein expression was significantly upregulated in the muscles, while SOD expression showed a trend toward increased levels. These changes in muscle antioxidants likely reflect adaptive mechanisms in response to exercise-induced stress. A similar protective effect of omega-3 FA against potential oxidative damage has been reported by Sarikaya et al. [87].

Several key limitations must be acknowledged in this study. Potential confounders in our study include the relatively small sample size per group, which may limit statistical power, variations in individual responses to the interventions, and the generalizability of our findings. Although two doses of omega-3 FAs were tested, exploring a broader range of doses or intervention durations could provide additional insights into dose–response relationships. The observed results, particularly the dose-dependent effects of omega-3 FAs, may require validation through larger-scale studies incorporating broader dose ranges and extended intervention periods. These limitations highlight the need for further research to elucidate the complex mechanisms associated with aging and evaluate the broader physiological impacts of these interventions. The molecular mechanisms underlying our observations require further investigation. Specifically, the role of the NRF2-Keap1 signaling pathway in mediating the antioxidant responses needs validation through targeted molecular approaches. Understanding this pathway’s activation patterns and downstream effects would provide mechanistic insights into how omega-3 FA supplementation and exercise modulate antioxidant enzyme expression. The current findings, while promising, represent preliminary evidence that requires systematic validation. Future investigations should focus on establishing the physiological impacts of combined omega-3 FA supplementation and exercise across both animal models and human subjects, particularly in the context of aging-related oxidative stress management.

## 5. Conclusions

In conclusion, this study investigated the potential synergistic effects of omega-3 FA supplementation and physical activity in 23–24-month-old rats. Physical activity alone positively influenced muscle growth, as indicated by the increased stride length and EDL muscle ratio, and modulated lipid oxidation within the muscles while enhancing antioxidant contributions in the blood. It also improved the levels of triacylglycerols. The combined intervention demonstrated dose-dependent effects, with the higher dose of omega-3 FA (320 mg/kg body weight) being more effective than the lower dose (160 mg/kg body weight). The higher dose significantly increased the plasma antioxidant capacity (TEAC) and tissue GPx protein expression, while SOD expression showed a trend toward upregulation. The observed decline in CAT activity in the erythrocytes may reflect adaptive responses to exercise-induced stress. Notably, combined interventions effectively normalized cholesterol levels within physiological ranges.

These findings highlight the complexity of the interactions between omega-3 FA supplementation, physical activity, and age-related physiological changes. Further research is essential to unravel the underlying mechanisms and evaluate the long-term benefits of such integrative approaches for aging populations, offering promising avenues for improving healthspan and combating age-related decline.

## Figures and Tables

**Table 1:** Functional, biochemical, and oxidative stress parameters in the sedentary and trained aged rats.

Parameters	Sedentary Aged Rats (*n* = 5)	Trained Aged Rats		
		Ex (*n* = 5)	O1 + Ex (*n* = 6)	O2 + Ex (*n* = 6)
Body weight (g)	526 (516; 587)	547 (490; 548)	546 (535; 561)	576 (550; 585)
Stride length (cm)	12.83 (11.25; 13.06)	14.02 (13.84; 14.16) *	14.48 (12.37; 15.36) *	14.73 (14.51; 14.94) ***
Muscle mass index (%) ^a^				
*Gastrocnemius*	0.393 (0.382; 0.414)	0.472 (0.418; 0.472)	0.478 (0.428; 0.491)	0.454 (0.425; 0.456)
*EDL*	0.035 (0.035; 0.035)	0.040 (0.039; 0.044) *	0.038 (0.033; 0.040)	0.036 (0.035; 0.037)
*Soleus*	0.038 (0.036; 0.041)	0.042 (0.041; 0.042)	0.041 (0.039; 0.044)	0.042 (0.042; 0.043)
Glucose (mmol/L)	6.60 (6.20; 7.00)	6.70 (6.70; 7.10)	6.60 (6.20; 6.85)	6.85 (6.50; 6.98)
Albumin (g/L)	30.6 (30.4; 31.2)	31.3 (30.8; 31.9)	31.7 (30.5; 32.6)	30.5 (30.2; 30.8)
Urea (mmol/L)	6.9 (6.7; 7.1)	6.7 (6.4; 7.6)	6.8 (6.6; 7.2)	7.3 (6.7; 7.9)
TC (mmol/L)	2.77 (2.45; 3.83)	2.74 (2.59; 2.74)	2.57 (2.27; 2.84)	2.54 (2.40; 2.60)
TAG (mmol/L)	1.59 (1.28; 2.02)	1.10 (1.10; 1.14) *	1.18 (0.90; 1.62)	1.25 (1.08; 1.41)
AOPP (µmol/L)	171.0 (170.7; 192.9)	159.2 (148.8; 178.6)	157.9 (152.7; 214.9)	175.9 (173.4; 183.1)
8-IsoP (pg/mL)	295.8 (195.8; 423.7)	190.1 (177.0; 357.7)	336.8 (248.3; 427.1)	347.9 (303.0;386.8)
LPx (nmol/mL)	102.2 (87.6; 112.1)	87.6 (78.2; 90.1)	94.8 (80.3; 124.3)	113.6 (101.6; 119.3)
SOD (U/mg Hb)	169.4 (162.7; 170.0)	169.7 (144.0; 179.7)	159.9 (149.6; 166.0)	170.5 (160.5; 189.5)
GPx (µkat/mg Hb)	3.62 (3.50; 3.90)	4.36 (4.20; 5.12) *	3.95 (3.35; 4.38)	4.01 (3.90; 4.06)
CAT (µkat/g Hb)	1.98 (1.84; 2.31)	2.16 (1.85; 2.26)	1.54 (1.37; 1.82) *	1.58 (1.43; 1.61) *
TEAC (µmol/L)	4.92 (3.22; 4.94)	5.59 (5.21; 6.26) *	5.27 (4.85; 5.68)	5.95 (5.50; 6.00) **
FRAP (µmol/L)	150.3 (148.1; 163.2)	112.9 (111.4; 127.7) *	119.8 (65.8; 142.1)	131.0 (112.6; 143.9)

Data are expressed as median and quartiles (Q1 and Q3). Statistically significant values are defined as *p* < 0.05. Comparison to the sedentary controls: * *p* < 0.05; ** *p* < 0.01; *** *p* < 0.001. Trained rats (Ex)—supplementation with a placebo daily for 3 weeks, followed by 4 weeks of placebo intake along with daily exercise for 10 min; O1 + Ex—supplementation with omega-3 fatty acids (FAs) in a dose of 160 mg/kg of body weight/daily for 3 weeks, followed by 4 weeks of omega-3 FA intake in the indicated dose with daily exercise for 10 min; O2 + Ex—supplementation with omega-3 FAs in a dose of 320 mg/kg of body weight/daily for 3 weeks, followed by 4 weeks of omega-3 FA intake in the indicated dose with daily exercise for 10 min. EDL—*extensor digitorum longus*; TC—total cholesterol; TAG—triacylglycerol; AOPP—advanced protein oxidation product; 8-IsoP—8-isoprostane; LPx—lipoperoxide; SOD—superoxide dismutase; GPx—glutathione peroxidase; CAT—catalase; TEAC—trolox equivalent antioxidant capacity; FRAP—ferric reducing antioxidant power; ^a^ muscle mass index was calculated as a ratio of weight of muscle concerned to total body weight.

**Table 2:** Effect of physical activity and combined intervention (omega-3 FA and physical activity at two different doses) on oxidative stress parameters in gastrocnemius muscle.

Parameters	Sedentary Aged Rats (*n* = 5)	Trained Aged Rats		
		Ex (*n* = 5)	O1 + Ex (*n* = 6)	O2 + Ex (*n* = 6)
TBARS (µmol/g proteins)	2.45 (2.10; 2.74)	1.73 (1.62; 1.86) *	1.97 (1.92; 1.99)	1.86 (1.57; 2.20)
TEAC (mmol/g proteins)	1.09 (1.06; 1.19)	1.16 (1.08; 1.16)	1.14 (1.08; 1.17)	1.06 (1.02; 1.11)
FRAP (µmol/g proteins)	11.04 (10.6; 12.11)	10.28 (10.21; 10.42)	10.55 (10.20; 11.50)	11.54 (10.10; 12.58)

Data are expressed as median and quartiles (Q1 and Q3). Statistically significant values are defined as *p* < 0.05. Comparison to the sedentary controls: * *p* < 0.05. Trained rats (Ex)—supplementation with a placebo daily for 3 weeks, followed by 4 weeks of placebo intake along with daily exercise for 10 min; O1 + Ex—supplementation with omega-3 fatty acids (FAs) in a dose of 160 mg/kg of body weight/daily for 3 weeks, followed by 4 weeks of omega-3 FA intake in the indicated dose with daily exercise for 10 min; O2 + Ex—supplementation with omega-3 FAs in a dose of 320 mg/kg of body weight/daily for 3 weeks, followed by 4 weeks of omega-3 FA intake in the indicated dose with daily exercise for 10 min; TBARS—thiobarbituric acid reactive substance; TEAC—trolox equivalent antioxidant capacity; FRAP—ferric reducing antioxidant power.

## Data Availability

The datasets generated and analyzed during the current study are not publicly available due to legal reasons, but are available from the corresponding author upon a reasonable request.
